# Supplementary material for: Customizable wave tailoring nonlinear materials enabled by bilevel inverse design
Source: Nat Commun. 2025 Apr 10;16:3425. doi: 10.1038/s41467-025-58630-8 (PMC11986126; doi:10.1038/s41467-025-58630-8)
Supplement: Supplementary file 1 — Supplementary Information [file 41467_2025_58630_MOESM1_ESM.pdf]

Supplementary Information: Customizable wave tailoring nonlinear materials enabled by bilevel inverse design

Brianna MacNider[1+] Haning Xiu[1+] Caglar Tamur[2] Kai Qian[1] Ian Frankel[1]
Maya Brandy[1] Hyunsun Alicia Kim[2,3] Nicholas Boechler[1,3,*]
[1]Department of Mechanical and Aerospace Engineering, University of California, San Diego, La Jolla, CA 92093, US
[2]Department of Structural Engineering, University of California, San Diego, La Jolla, CA 92093,US
[3]Program in Materials Science and Engineering, University of California, San Diego, La Jolla, CA 92093, US

Note 1: Two and three dimensional nonlinear mesostructured material analogs

In Fig. S1, we show the concepts for two- and three-dimensional (2D and 3D) generalization of the optimized nonlinear mesostructured material described in the main text. We note that relative transverse and rotation between the masses, in addition the longitudinal translation described in the main text, would need to be accounted for for in any design optimized for such higher dimensions (which has not been considered in this work).

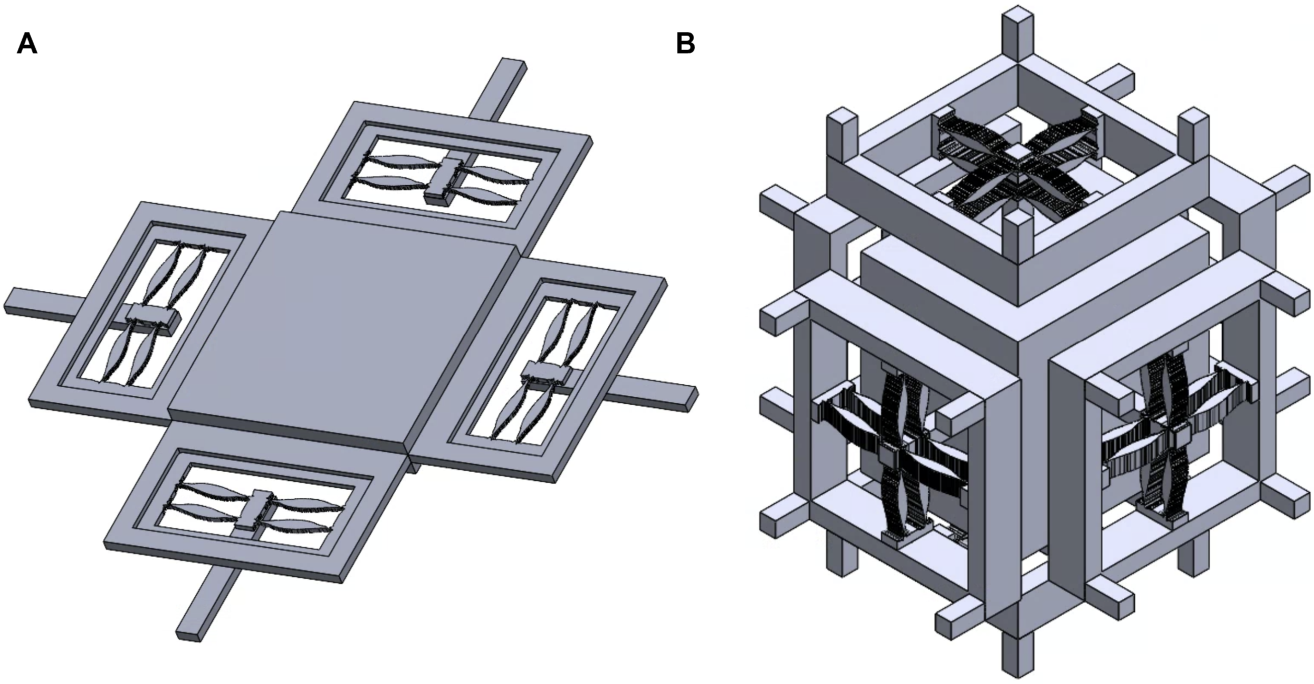

Figure S1: A) 2D generalization of an optimized nonlinear mesostructured material. B) 3D generalization of an optimized nonlinear mesostructured material.

Note 2: Fifth order springs

Here we consider a nonlinear springs with a fifth order polynomial to govern its force-displacement relation, $f(\Delta x) = \Delta x + c_2(\Delta x)^2 + c_3(\Delta x)^3 + c_4(\Delta x)^4 + c_5(\Delta x)^5$. Considering deformations in the range $\Delta x \in [0, 1]$, the positive strain energy condition becomes

$$P(\Delta x) = \int_0^1 f(\Delta x) d\Delta x > 0 \; , \tag{S.1}$$

which leads to a constraint on the polynomial coefficients

$$30 + 20c_2 + 15c_3 + 12c_4 + 10c_5 > 0 \ . \tag{S.2}$$

The material system is simulated via DEM with $N = 20$ particles in the chain, with damping $\zeta = 0.01$ and with the impact conditions $M/M_0 = 0.05$ and $V/V_0 = 1$. For the optimization, the objective is chosen as the peak kinetic energy at the last particle, normalized by the linear response, i.e., $\max(KE_{non})/\max(KE_{lin})$. A gradient based optimizer is used to minimize this objective, while constraining the design space to satisfy the positive energy condition, Eq. S.2. The best nonlinearity obtained from the optimization is $f(\Delta x) = \Delta x - 4.71\Delta x^2 + 1.97\Delta x^3 + 17.74\Delta x^4 - 15.05\Delta x^5$, which results in a performance of $\max(KE_{non})/\max(KE_{lin}) = 3.8\%$. The resultant optimized spring and the corresponding spatiotemporal KE response are shown in Fig. S2. The fifth order optimized springs display qualitative and quantitative similarity to the cubic polynomial springs discussed in the main text within the current deformation range; both having snap-through mechanisms and achieving comparable dynamic performances, thus demonstrating the adequacy of cubic polynomials for KE minimization.

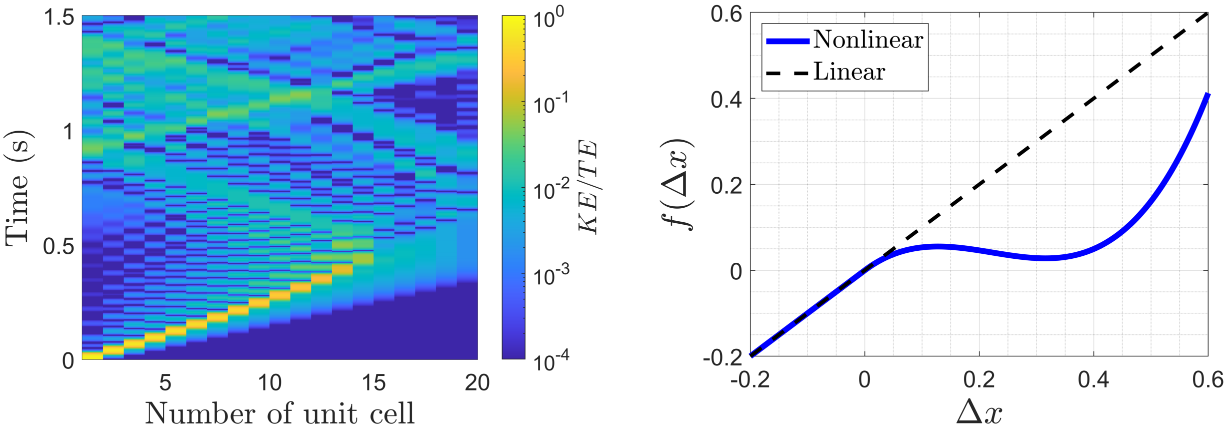

Figure S2: Results of optimized 5th order springs. (a) Spatiotemporal kinetic energy response. (b) Force-displacement relations of springs. Source data are provided as a Source Data file.

Note 3: Reduced order discrete element model and non-dimensionalization

Our reduced order discrete element model (DEM) of a nonlinear material (Fig. S3) includes N layers of unit cells. Each unit cell consists of a lumped mass m, a massless spring $f^*(\Delta x^*)$ where Δx^* is the spring stretch such that Δx^* and f^* are postitive in tension, and a linear inter-site viscous damper η^*. The equation of motion of the ith unit cell can be written as

$$m\ddot{x}_i^* + f^*(x_i^* - x_{i-1}^*) - f^*(x_{i+1}^* - x_i^*) + \eta^*(-\dot{x}_{i+1}^* + 2\dot{x}_i^* - \dot{x}_{i-1}^*) = 0, \tag{S.3}$$

where x_i^* is the displacement of the ith particle from its rest position.

In this work, we consider cubic polynomial nonlinear springs in compression and linear springs in tension. Considering first then the compressional behavior, e.g., $x_{i+1}^* \leq x_i^*$ and $x_i^* \leq x_{i-1}^*$, Eq. (S.3) can be replaced by

$$\begin{aligned} &m\ddot{x}_i^* - c_1^*(x_{i+1}^* - x_i^*) + c_2^*(x_{i+1}^* - x_i^*)^2 - c_3^*(x_{i+1}^* - x_i^*)^3 \\ &+ c_1^*(x_i^* - x_{i-1}^*) - c_2^*(x_i^* - x_{i-1}^*)^2 + c_3^*(x_i^* - x_{i-1}^*)^3 + \eta^*(-\dot{x}_{i+1}^* + 2\dot{x}_i^* - \dot{x}_{i-1}^*) = 0. \end{aligned} \tag{S.4}$$

We note that the coefficient c_2^* is opposite sign from c_1^* and c_3^*, which we choose for convenience. The overdot on the variables with the * denotes the time derivative, such that $\dot{x}^* = dx^*/dt^*$.

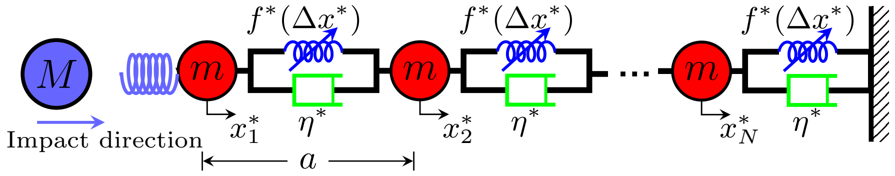

Figure S3: Schematic of the DEM. Horizontal arrows on the masses denote positive x^*.

Next, all variables and parameters are normalized by the length of a unit cell, a, the mass of a unit cell, m, and/or the linear stiffness of the spring, c_1^*, as follows

$$\begin{aligned} &x_i = \frac{x_i^*}{a}, \quad t = \frac{t^*}{\sqrt{m/c_1^*}}, \quad \frac{dx_i}{dt} = \frac{\sqrt{m/c_1^*}}{a}\frac{dx_i^*}{dt^*}, \quad \frac{d^2x_i}{dt^2} = \frac{m}{c_1^* a}\frac{d^2x_i^*}{dt^{*2}}, \\ &c_2 = \frac{c_2^* a}{c_1^*}, \quad c_3 = \frac{c_3^* a^2}{c_1^*}, \quad \zeta = \frac{\eta^*}{2\sqrt{mc_1^*}}, \end{aligned} \tag{S.5}$$

and the linear sound speed is $V_0 = a\sqrt{c_1^*/m}$.

The nondimensional equation of motion of the ith unit cell is then given by

$$\begin{aligned} &\ddot{x}_i - (x_{i+1} - x_i) + c_2(x_{i+1} - x_i)^2 - c_3(x_{i+1} - x_i)^3 \\ &+ (x_i - x_{i-1}) - c_2(x_i - x_{i-1})^2 + c_3(x_i - x_{i-1})^3 + 2\zeta(-\dot{x}_{i+1} + 2\dot{x}_i - \dot{x}_{i-1}) = 0. \end{aligned} \tag{S.6}$$

The overdot on variables without the * denotes the derivative with respect to non-dimensional time, such that $\dot{x} = dx/dt$.

The impactor, with mass of M and initial velocity of V, is assumed to interact with the nonlinear material via a contact spring. A contact spring featuring nonlinear stiffness, positioned between the first unit cell and the impactor mass, has been incorporated into the model to allow smooth contact and free release upon rebound. The contact spring is a Hertzian-like [1] nonlinear contact spring of the form

$$F_H^*(x_0^*, x_1^*) = C_{imp}[x_0^* - x_1^*]_+^{3/2}, \tag{S.7}$$

where the $[\cdot]_+$ denotes that the quantity in between the brackets only takes positive values and equals zero when negative, and F_H^* is positive when in compression. The provision of compressed force between the impactor mass and the material through bouncing force occurs only if $x_0^* > x_1^*$. The non-dimensional contact force is

$$F_H(x_0, x_1) = \frac{F_H^*(x_0^*, x_1^*)}{c_1^* a} \tag{S.8}$$

such that

$$F_H(x_0, x_1) = \frac{C_{imp}\sqrt{a}}{c_1^*}[x_0 - x_1]_+^{3/2}. \tag{S.9}$$

The entire nondimensional DEM of the impact dynamics system consisting of an N-layered nonlinear metamaterial is thus

$$\begin{cases} m_{im}\ddot{x}_0 + F_H(x_0, x_1) = 0 \quad (x_0 > x_1) \\ \ddot{x}_i - (x_{i+1} - x_i) + c_2(x_{i+1} - x_i)^2 - c_3(x_{i+1} - x_i)^3 + 2\zeta(-\dot{x}_{i+1} + \dot{x}_i) - F_H(x_0, x_1) = 0 \quad (i = 1) \\ \ddot{x}_i - (x_{i+1} - x_i) + c_2(x_{i+1} - x_i)^2 - c_3(x_{i+1} - x_i)^3 \\ +(x_i - x_{i-1}) - c_2(x_i - x_{i-1})^2 + c_3(x_i - x_{i-1})^3 + 2\zeta(-\dot{x}_{i+1} + 2\dot{x}_i - \dot{x}_{i-1}) = 0 \quad (2 \le i \le N-1) \\ \ddot{x}_i + x_i - c_2 x_i^2 + c_3 x_i^3 + (x_i - x_{i-1}) + c_2(x_i - x_{i-1})^2 + c_3(x_i - x_{i-1})^3 + 2\zeta(2\dot{x}_i - \dot{x}_{i-1}) = 0 \quad (i = N), \end{cases} \tag{S.10}$$

where $m_{im} = M/m$, and Eq. (S.10) is valid when the springs are in compression. When the springs are in tension, we set $c_2 = 0$ and $c_3 = 0$.

Note 4: Fully nonlinear springs

In this section we consider a fully nonlinear spring, which is governed by a third-order polynomial in both tensile and compressive deformations, as opposed to the linear tensile regime in the main text. The force-displacement relationship has the usual form $f(\Delta x) = \Delta x + c_2(\Delta x)^2 + c_3(\Delta x)^3$ and we consider deformations $\Delta x \in [-1, 1]$. To satisfy the positive strain energy for tensile and compressive deformations, we have

$$P^+(\Delta x) = \int_0^1 f(\Delta x) d\Delta x > 0 \quad \text{and} \quad P^-(\Delta x) = \int_0^{-1} f(\Delta x) d\Delta x > 0 \ , \tag{S.11}$$

which results in two constraints on the coefficients

$$c_2 > -\frac{3}{2} - \frac{3c_3}{4} \quad \text{and} \quad c_2 < \frac{3}{2} + \frac{3c_3}{4} \ . \tag{S.12}$$

We conduct DEM simulations on the same system that is described in the main text, with $N = 20$ particles in the chain and $\zeta = 0.01$ and with the impact conditions $M/M_0 = 0.05$ and $V/V_0 = 1$. To investigate the effect of fully nonlinear springs, we start by a preliminary exploration of the parameter space $c_2 \in [-20, 10]$ and $c_3 \in [0, 100]$. The results are reported in terms of the maximum kinetic energy at the end of the material, normalized by the corresponding linear system, as shown in Fig. S4(a), followed by a finer search in the sensitive region shown in Fig. S4(b).

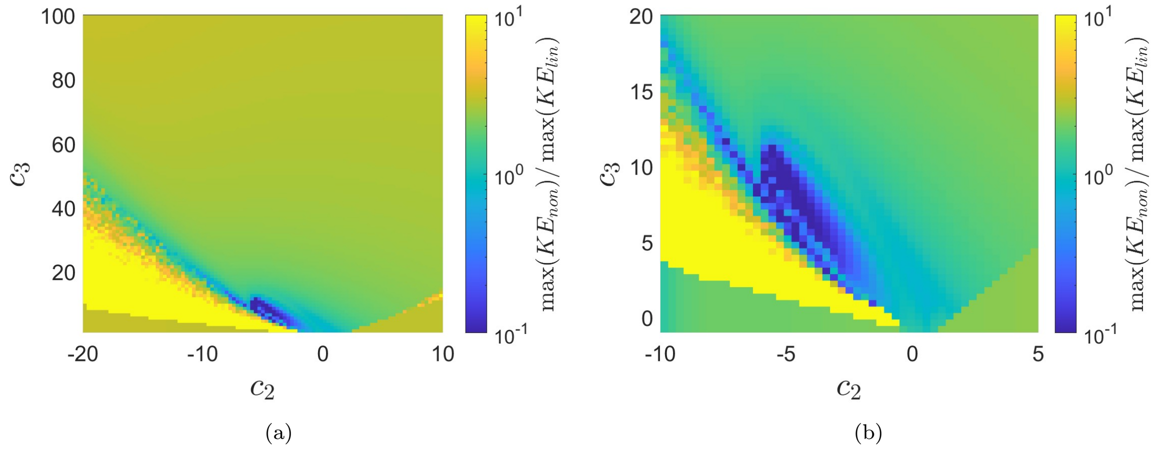

Figure S4: Normalized maximum kinetic energy at the last particle of the chain a function of nonlinear spring coefficients with the fully nonlinear springs. (a) Initial design space. (b) Sensitive region. Source data are provided as a Source Data file.

The gradient based optimization approach is applied to the problem to find the optimal spring coefficients by minimizing the KE ratio with using the constraints given in Eq. (S.12) and limiting the design space into the sensitive region in Fig. S4(b). The best spring is found to be $f(\Delta x) = \Delta x - 5.70\Delta x^2 + 10.54\Delta x^3$ with a KE ratio of $\max(KE_{non})/\max(KE_{lin}) = 3.8\%$, which is comparable to the results discussed in the main text. The resultant KE transmission is shown in Fig. S5(a) with the corresponding optimal spring in Fig. S5(b). We conclude that we have not observed a significant difference between using a fully nonlinear spring versus the one with a linear tensile regime in the context of KE minimization.

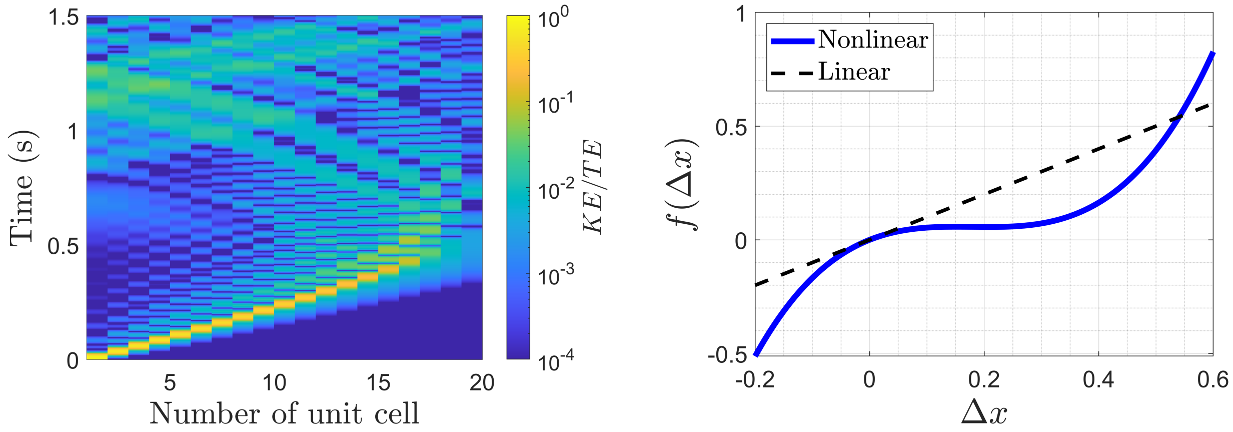

(a) (b)

Figure S5: Results of optimized fully nonlinear springs. (a) Spatiotemporal kinetic energy response. (b) Force displacement relations of springs. Source data are provided as a Source Data file.

Note 5: Characteristics of the nonlinear spring (3rd order polynomial)

The non-dimensional nonlinear spring in this study is chosen to conform to a third-order polynomial representation, expressed as $f(\Delta x) = f^*(\Delta x^*)/c_1^* a = \Delta x + c_2(\Delta x)^2 + c_3(\Delta x)^3$. In this section, we describe $\Delta x \in [0, 1]$ for simplicity, despite compression being considered in the main text. For compression ($\Delta x < 0$) the only thing that changes from the analysis is that the sign of c_2 is flipped for $\Delta x < 0$. We also set $c_3 > 0$ for simplicity. By manipulating both the sign and magnitude of c_2, the function $f(\Delta x)$ exhibits the potential for either two extrema (a local maximum and a local minimum), a single extremum (local maximum), or none at all (Fig. S6). We consider each case further in the following.

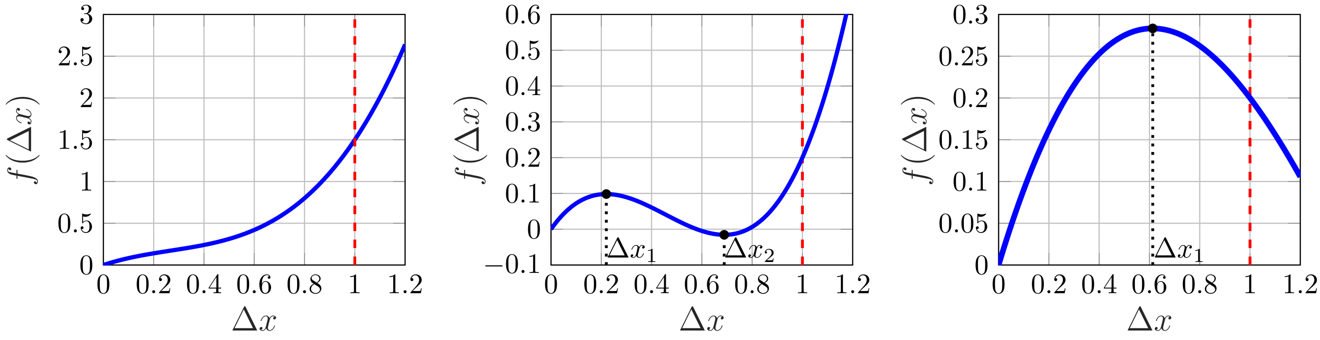

(a) (b) (c)

Figure S6: Force-displacement characteristics of third-order polynomial nonlinear springs that exhibit the following behaviors: (a) monotonic increment, (b) bistability/snap-through, and (c) local maximum in the function $f(\Delta x)$. Source data are provided as a Source Data file.

5.1 Monotonic increase of $f(\Delta x)$

For monotonic increase, we must satisfy the condition

$$f'(\Delta x) \geq 0, \tag{S.13}$$

where $f' = df/d\Delta x$, such that

$$1 + 2c_2\Delta x + 3c_3(\Delta x)^2 \geq 0. \tag{S.14}$$

For our considered range of parameters, Eq. (S.14) is always satisfied if

$$c_2 > -\sqrt{3c_3}, \tag{S.15}$$

which corresponds to the cases of no real roots or $c_2 \geq 0$.

An additional condition for ensuring a monotonic increase in the function $f(\Delta x)$ for $\Delta x \in [0, 1]$ is that the local maximum of $f(\Delta x)$ should be situated at $\Delta x \geq 1$. In such a case, the small-valued root Δx_1 of $f'(\Delta x) = 0$ must satisfy

$$\Delta x_1 \geq 1 \tag{S.16}$$

where

$$\Delta x_1 = \frac{-c_2 - \sqrt{c_2^2 - 3c_3}}{3c_3}. \tag{S.17}$$

For real Δx_1 (Eq. (S.17)), the coefficients must thus satisfy

$$c_2 \leq -\sqrt{3c_3} \tag{S.18}$$

and

$$-\sqrt{c_2^2 - 3c_3} \geq c_2 + 3c_3. \tag{S.19}$$

From Eq. (S.19), we obtain the criteria

$$c_2 \leq -3c_3 \tag{S.20}$$

and

$$c_2 \geq -\frac{1 + 3c_3}{2}. \tag{S.21}$$

Considering Eqs. (S.15), (S.18), (S.20) and (S.21), we can then say that $f(\Delta x)$ exhibits a monotonic increase when the coefficients satisfy: i) $c_2 > -\sqrt{3c_3}$; or ii) $c_2 \leq -\sqrt{3c_3}$, $c_2 \leq -3c_3$, and $c_2 \geq -(1 + 3c_3)/2$ simultaneously.

5.2 Bistability or snap-through of $f(\Delta x)$

To establish a bistable curve, inclusive of snap-through behavior, it is necessary that the local maxima and minima both occur within the interval of $\Delta x \in (0, 1)$. The strain (or potential) energy must also remain positive in this same interval. To satisfy the first criterion, the roots of $f'(\Delta x) = 0$

$$\Delta x_{1,2} = \frac{-c_2 \pm \sqrt{c_2^2 - 3c_3}}{3c_3} \tag{S.22}$$

must satisfy

$$0 < \Delta x_{1,2} < 1. \tag{S.23}$$

As before, to obtain real roots

$$c_2 \leq -\sqrt{3c_3} \tag{S.24}$$

must be satisfied. As $\sqrt{c_2^2 - 3c_3} \geq 0$, Eq. (S.23) can be rewritten as

$$-c_2 - \sqrt{c_2^2 - 3c_3} > 0, \tag{S.25}$$

and

$$-c_2 + \sqrt{c_2^2 - 3c_3} < 3c_3. \tag{S.26}$$

Equation (S.25) is always satsified for $c_2 \leq -\sqrt{3c_3}$. Equation (S.26) generates the two conditions

$$c_2 > -3c_3, \tag{S.27}$$

and

$$c_2 > -(1 + 3c_3)/2. \tag{S.28}$$

Next, to satisfy the condition of potential energy $P(\Delta x) > 0$ we define

$$P(\Delta x) = \int_0^1 f(\Delta x) d\Delta x \tag{S.29}$$

which gives the condition

$$\frac{1}{2} + \frac{c_2}{3} + \frac{c_3}{4} > 0 \tag{S.30}$$

that simplifies to

$$c_2 > -\frac{3}{2} - \frac{3c_3}{4}. \tag{S.31}$$

Combining Eqs. (S.24), (S.27), (S.28), and (S.31), when the coefficients satisfy $c_2 > \max[-(1+3c_3)/2, -3c_3, -3/2 - 3c_3/4]$, and $c_2 \leq -\sqrt{3c_3}$, the function $f(\Delta x)$ can exhibit bistability/snap-through.

5.3 Local maximum of $f(\Delta x)$ for $\Delta x \in (0, 1)$

When the function only has a local maximum within $\Delta x \in (0, 1)$ and no local minimum, we should ensure $0 < \Delta x_1 < 1$ and $\Delta x_2 > 1$, such that

$$0 < \frac{-c_2 - \sqrt{c_2^2 - 3c_3}}{3c_3} < 1, \tag{S.32}$$

and

$$\frac{-c_2 + \sqrt{c_2^2 - 3c_3}}{3c_3} > 1. \tag{S.33}$$

Equation (S.32) gives the conditions

$$c_2 \leq -\sqrt{3c_3}, \tag{S.34}$$

and

$$c_2 < -\frac{1+3c_3}{2}. \tag{S.35}$$

Equation (S.33) can be rewritten as

$$\sqrt{c_2^2 - 3c_3} > c_2 + 3c_3. \tag{S.36}$$

If $c_2 + 3c_3 \leq 0$, Eq. (S.36) is always satisfied, and results in the condition

$$c_2 \leq -3c_3. \tag{S.37}$$

On the other hand, if $c_2 + 3c_3 > 0$, we must have

$$c_2 < -\frac{1+3c_3}{2} \tag{S.38}$$

to satisfy Eq. (S.36), which is equivalent to Eq. (S.35).

Considering Eqs. (S.34), (S.35), (S.37) and (S.31), the parameters thus need to satisfy

$$\begin{aligned} &c_2 > -\frac{3}{2} - \frac{3c_3}{4} \quad \text{and} \\ &c_2 < \min\left(-\frac{1+3c_3}{2}, -\sqrt{3c_3}, -3c_3\right), \end{aligned} \tag{S.39}$$

or

$$\begin{aligned} &c_2 > \max\left(-\frac{3}{2} - \frac{3c_3}{4}, -3c_3\right) \quad \text{and} \\ &c_2 < \min\left(-\frac{1+3c_3}{2}, -\sqrt{3c_3}\right). \end{aligned} \tag{S.40}$$

Equation (S.40), however, lacks a valid set. As a result, the occurrence of a single local maximum for $f(\Delta x)$ within $0 < \Delta x < 1$ is contingent upon satisfying Eq. (S.39).

Note 6: Self-localization in the optimal nonlinear system for minimizing peak transmitted kinetic energy

In Fig. S7, we examine the pulse profiles in our system at different time intervals to illustrate the localization phenomena occuring in the nonlinear, optimal system. For the linear system (Fig. S7(b)), the pulse exhibits a broader width, with an oscillating tail that grows in time, which is consistent with linear dispersive effects. In contrast, the pulses in the nonlinear material exhibit narrower widths and approximately retain their shape as it propagates, which is consistent with solitary wave behavior. A slight decay can be seen for both the linear and nonlinear systems, which can be attributed to the small level of damping present in the system, and in the linear case, dispersive effects (assuming the nonlinearity is balancing the dispersion in the nonlinear case).

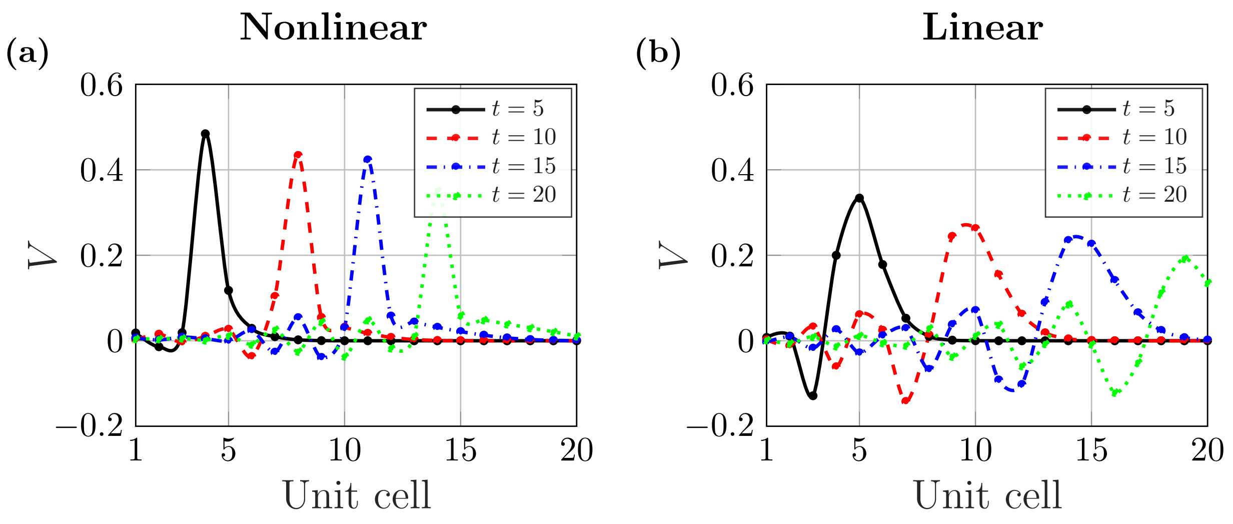

Figure S7: Spatial profiles of solitary waves at different dimensionless time steps in the (a) linear and (b) nonlinear systems from Fig. 2D and 2F. Source data are provided as a Source Data file.

Note 7: Additional DEM results for minimizing peak transmitted kinetic energy

Figure S8 illustrates the best KE ratios at the end of the material and their corresponding nonlinear spring coefficients for varied impactor mass and velocities ($N = 20$, $\zeta = 0.025$). Figure S9 shows the best KE ratios at the mid-point of the material and the corresponding coefficients for a 100-unit-cell chain and $\zeta = 0.025$.

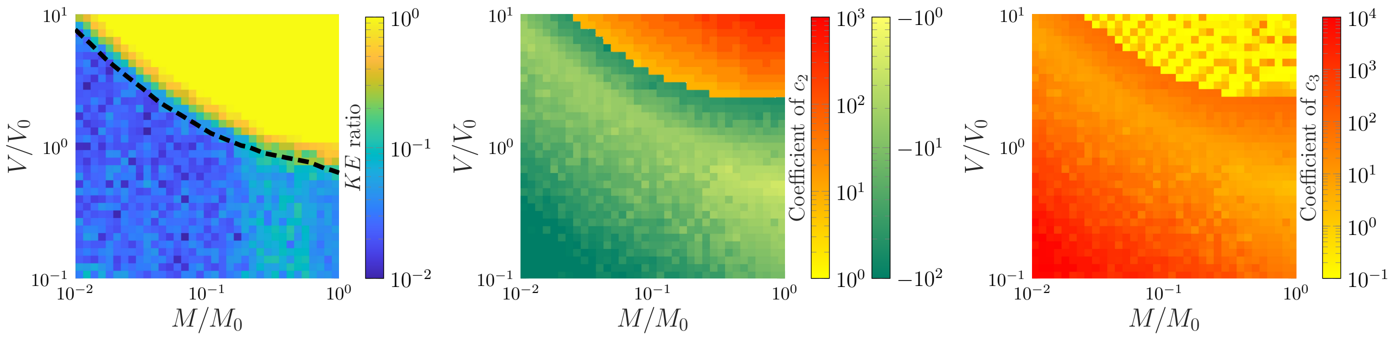

(a) (b) (c)

Figure S8: (a) KE ratio at the end of the material with respect to impactor mass and velocity and corresponding nonlinear spring coefficients (b) c_2 and (c) c_3 for a energy absorbing material with $N = 20$, $\zeta = 0.025$. The impactor mass and velocity are swept from $0.01M_0$ to M_0, and $0.1V_0$ to $10V_0$, respectively. Source data are provided as a Source Data file.

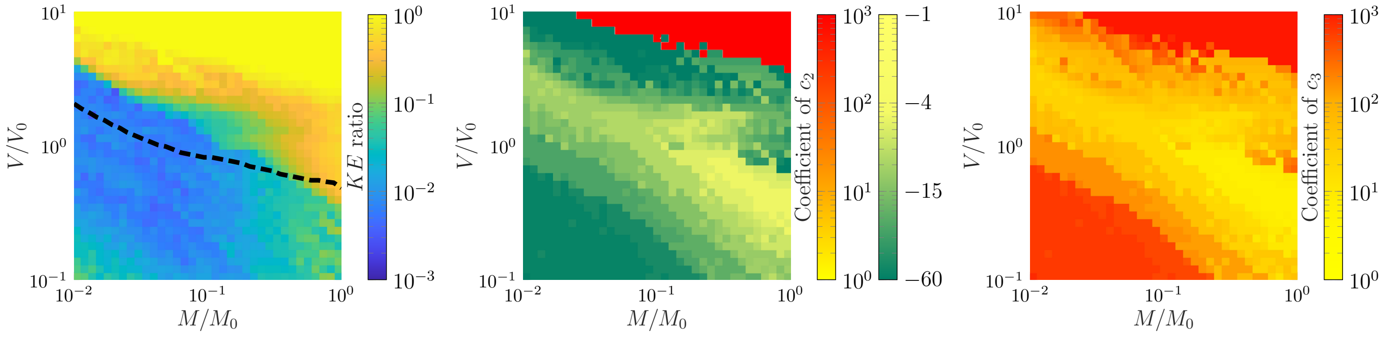

(a) (b) (c)

Figure S9: (a) KE ratio at the middle of the material as a function of impactor mass and velocity and corresponding nonlinear spring coefficients (b) c_2 and (c) c_3 for a energy absorbing material with $N = 100$, $\zeta = 0.025$. The impactor mass and veloscity are swept from $0.01M_0$ to M_0, and $0.1V_0$ to $10V_0$, respectively. Source data are provided as a Source Data file.

Note 8: Unit cell design and damping characterization

The initial condition used to optimized the unit cell mesostructure is depicted in Fig. S10. The boundary conditions imposed are also depicted — fixed along the top edge, rollers along the bottom edge (allowing for a symmetric reflection of the structure across the bottom edge for a two spring unit cell), with an applied displacement across the right edge, from 0 to 0.1 of the domain length (a region defined by $x = 1$, $y = 0$ to 0.1).

The top edge fixed boundary is particularly important for the bistable design, as the transition from one stable state to another requires it to pass through a compressive deformation that presses against both the top and bottom edges. If the frame housing the optimized spring is not strong enough relative to the spring to impose a sufficiently rigid boundary, the bistability of the response is reduced. Accordingly, the frame (as shown in Fig. 5C in the main text) for each spring was designed to be higher modulus and thicker than the spring itself, such that boundary conditions would be sufficiently rigid to enable the nonlinear response. This was confirmed via FEM simulation prior to manufacture, and via quasi-static experiment (see Fig. 5D in the main text) once the design had been manufactured.

The spring used for the linear chain is shown in Fig. S11. The final manufactured design is shown on the left panel of the figure. We note that four individual linear springs were used in the final design. More springs were added to increase the stiffness of the linear design such that it would be closer to that of the nonlinear design. The final

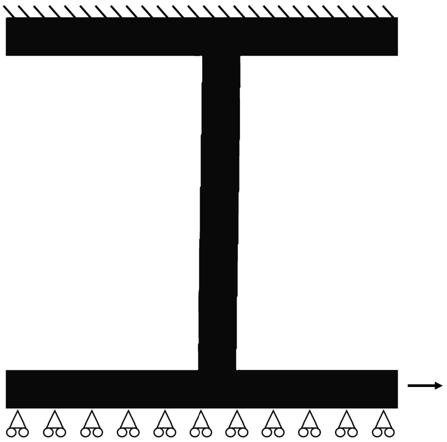

Figure S10: Initial condition for the topology optimization of the nonlinear spring.

experimental linear stiffness of the nonlinear spring was found to be 1.0598 kN/m, while the linear spring was found to be 1.0454 kN/m, both as measured from quasi-static tests. The green marker used to track unit cell motion is visible on the right edge of the manufactured unit cell. The quasi-static test of the linear spring can be seen in Fig. S12.

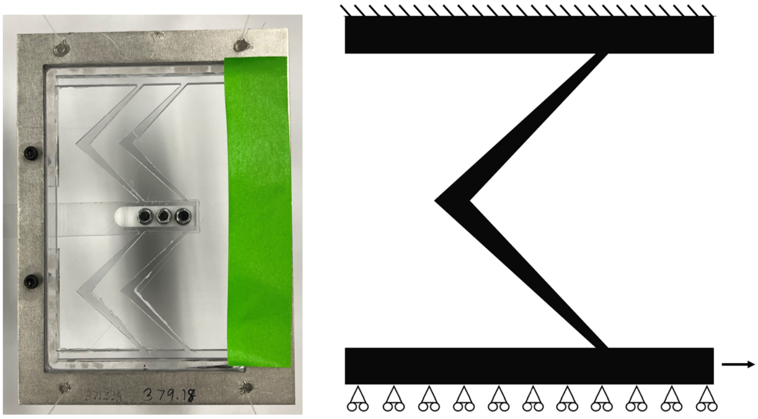

Figure S11: Design of the linear spring used as the control for comparison with the optimized nonlinear spring. The right panel shows the design of a single linear spring, including the boundary conditions. The left panel shows the final manufactured linear spring unit cell, along with the green marker used for image tracking in the experiment videos on the right edge.

As mentioned in the main text, there was some concern about the onset of plasticity and the effects on the optimized structure of repeated loading due to multiple experimental trials. Multiple cycles of quasi-static loading tests were therefore conducted to confirm that the response of the structure did not appreciably degrade over multiple loading cycles. The results of this testing are shown in Fig. S13. Five tests were conducted to just over half the maximum designed range of displacement (4 mm), and five were conducted up to 7 mm. We note that the spring was pressed slightly down while fixing it into the load frame, and is therefore not necessarily beginning from the precise equilibrium state in theses tests, but this should have no bearing on the intented purpose (which was to confirm no onset of plasticity).

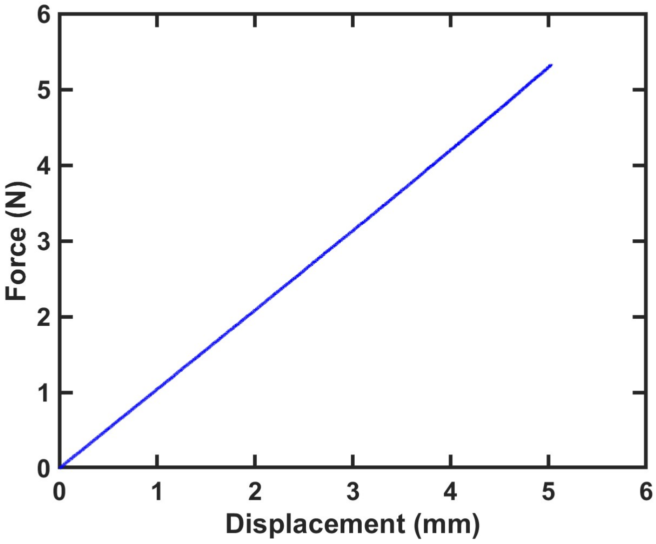

Figure S12: Quasi-static experimental test of the manufactured linear spring, confirming stiffness and linearity of behavior. Source data are provided as a Source Data file.

The plots associated with the damping characterization, described in the main text in the Methods section, are included here. Fig. S14(a) and (b) show the experimental setup. Measured dynamical responses, which were processed to account for the tilted angle between the LDV scanning head and the unit cell, are shown in Fig. S15(a) and (c). The normalized power spectrum is shown in Fig. S15(b) and (d).

In Fig. S16, we show the simulated effect of differences in damping on KE ratio. The two damping values considered are the experimentally characterized damping ratio for the linear chain ($\zeta = 0.003$) compared to a damping ratio for the linear chain of just larger than that measured for the nonlinear chain ($\zeta = 0.006$). This level of difference in damping can be seen to have minimal effect on the performance metric, confirming the dominant effect of varied nonlinearity.

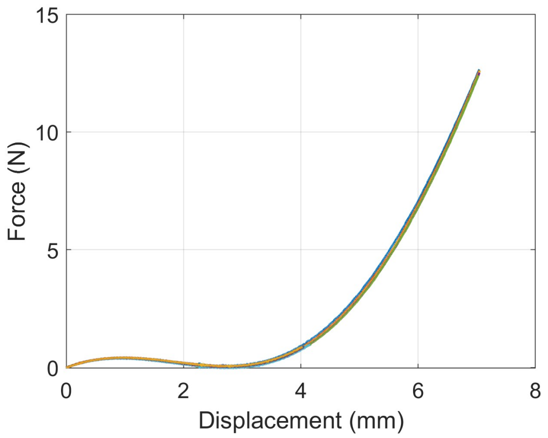

Figure S13: Results of quasi-static cyclic loading testing. A total of 10 tests were run, with tests 1 through 5 being run to a maximum displacement partially through the expected displacement range, and tests 6 through 10 sweeping through the entire expected operating displacement range of the structure. Source data are provided as a Source Data file.

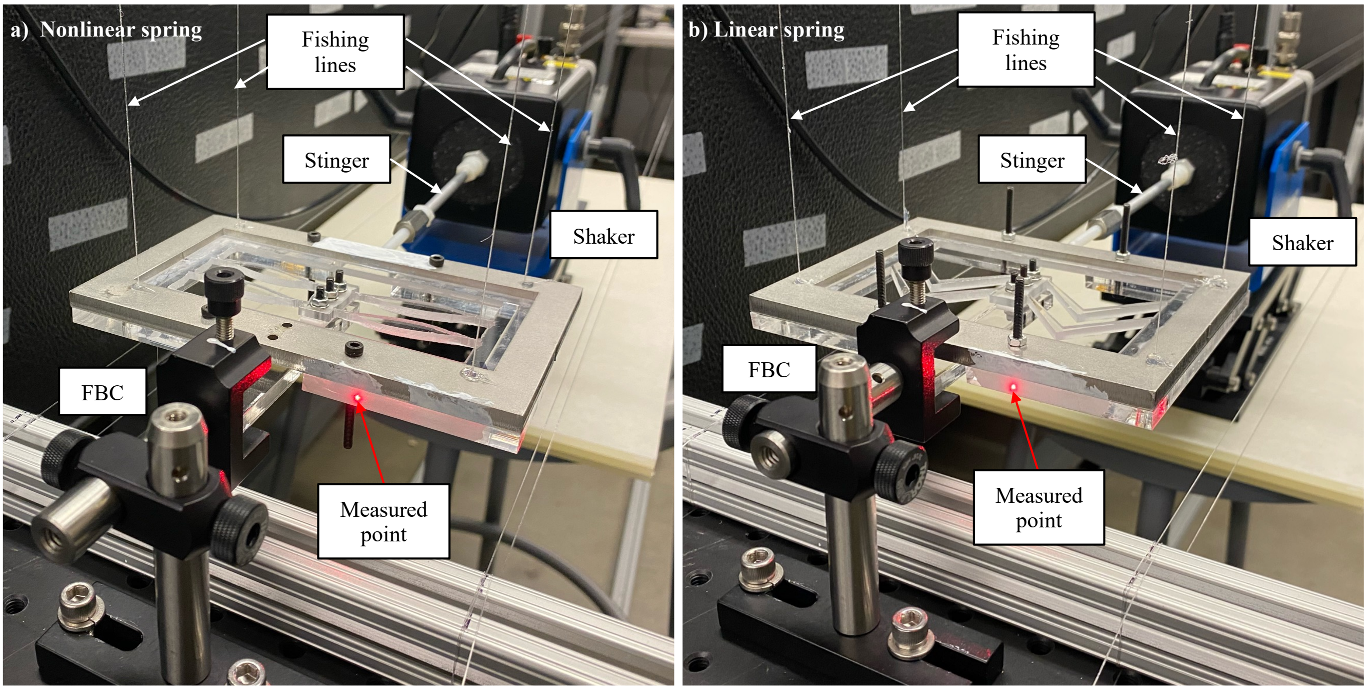

Figure S14: Experimental setup for characterizing the damping ratio of a single unit cell for (a) nonlinear and (b) linear chains. LDV (not appear in the photos) was placed behind the metal clamp that serves as a fixed boundary condition (FBC). The tilted angle of the LDV has been considered and processed in the data analysis.

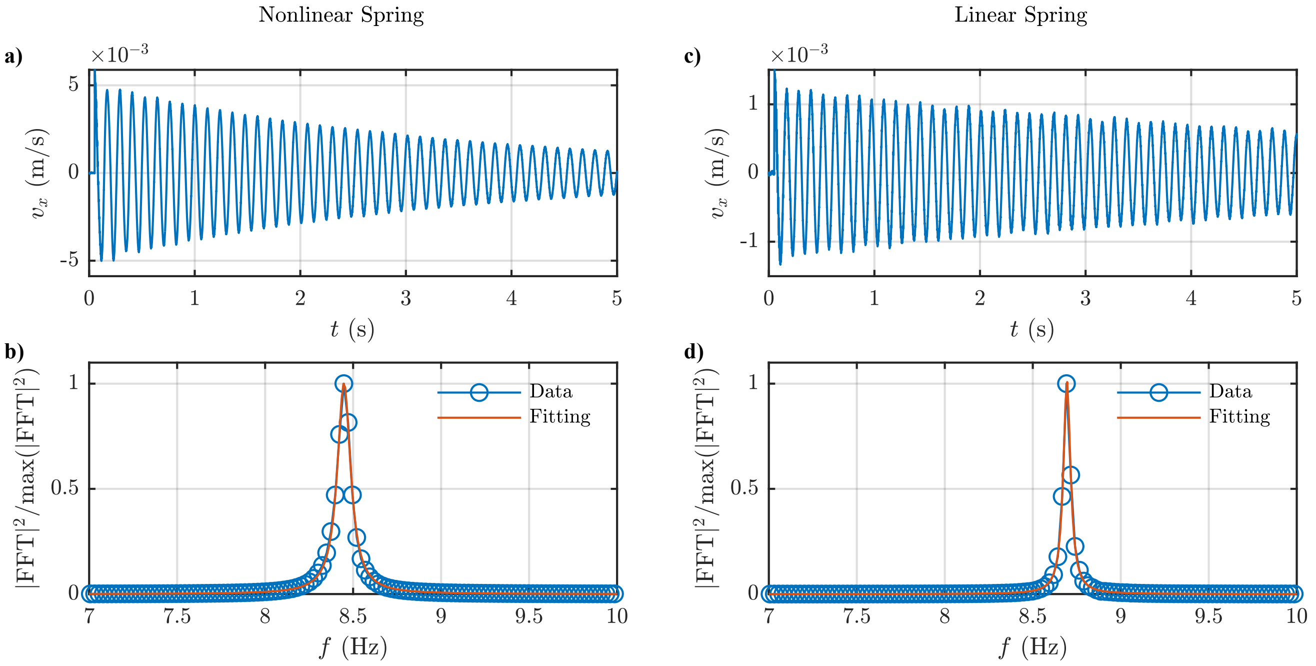

Figure S15: Experimental results for characterizing the damping ratio of a single unit cell for (a-b) nonlinear and (c-d) linear chains. (a,c) Processed time domain data (first 5 s), where the pulse excitation was applied at 20 ms. (b,d) Normalized power spectrum of the entire time domain data (about 41 s) with the fitting line of the Lorentzian function. Source data are provided as a Source Data file.

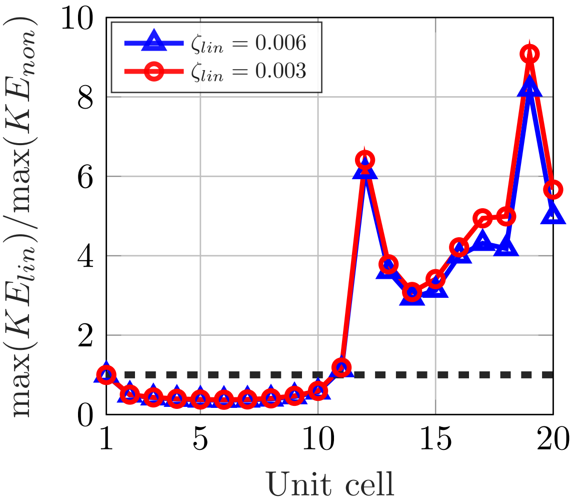

Figure S16: The maximum kinetic energy (linear/nonlinear) seen at each unit cell for simulation with different linear damping ratios. The nonlinear spring is fixed with $\zeta = 0.005$, and the linear spring has damping ratios of $\zeta_{lin} = 0.006$ and $\zeta_{lin} = 0.003$. Source data are provided as a Source Data file.

Note 9: Additional experimental details and results

A more thorough view of the experimental setup, comparing the experimental system to the DEM, is presented in Fig. S17. Further details of the unit cell and connectors are shown in Figure S18.

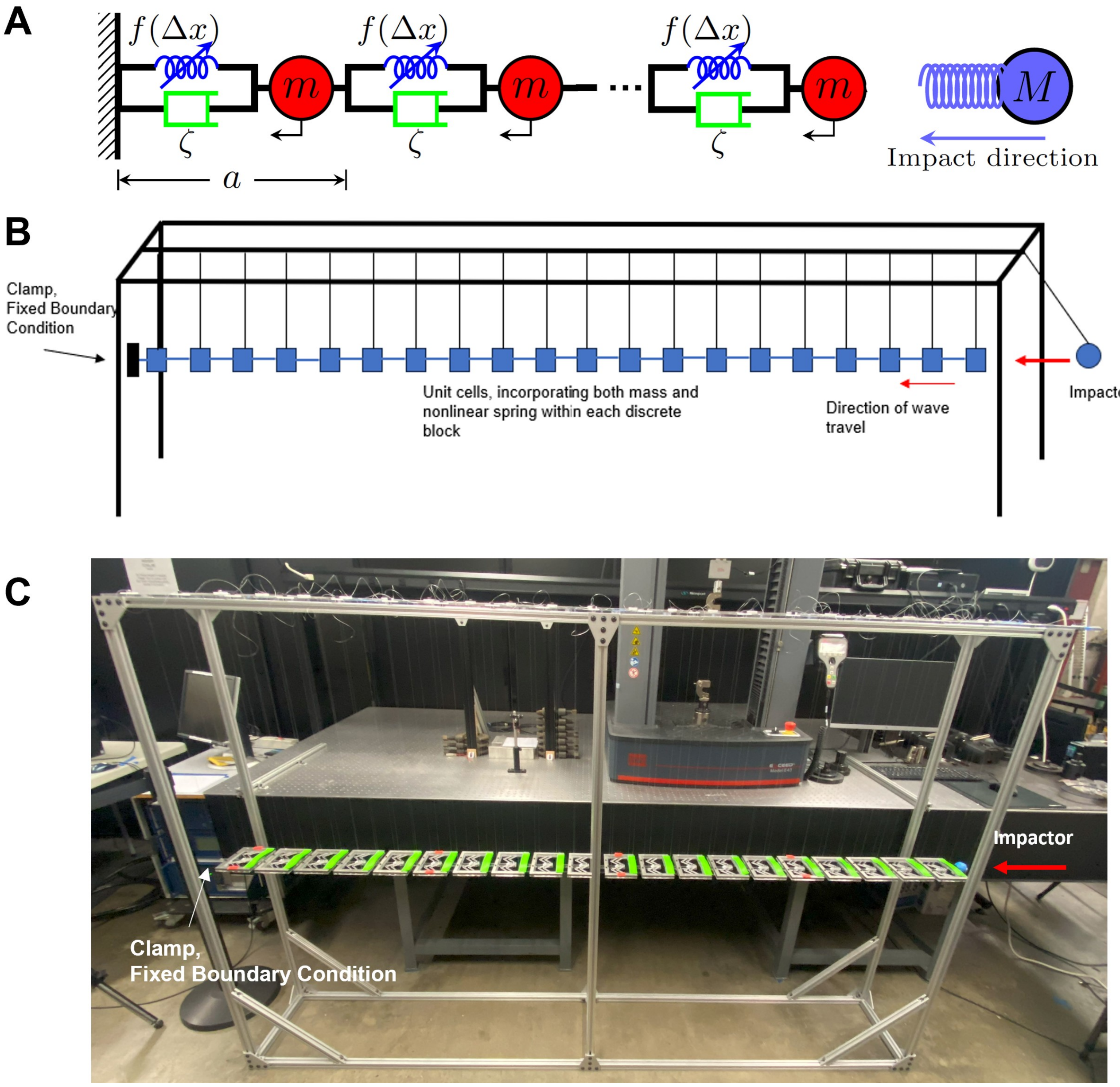

Figure S17: A more detailed breakdown of the experimental setup described in the main text. A) The discrete model used to simulate the system. B) A sketch of the experimental system, analogous to the model shown in A. The system is clamped on the left to impose a fixed boundary condition, while the imapactor collides with the system on the right. C) A photograph of the actual experimental system, analogous with the portions in A and B. The chain shown here is the linear chain, however, we note that the setup is the same for both the nonlinear and linear chains (only the design of the unit cell varies). Note that the clamp on left edge used to impose the fixed boundary is not attached in the photo.

The 24 videos associated with the experiments (4 each for 3 nonlinear experiments and 3 linear experiments) are included in the following data repository: Boechler, Nicholas (2024), "Customizable wave tailoring materials enabled by nonlinear bilevel inverse design 1", Mendeley Data, V1, doi: 10.17632/2wgwfy2wfg.1. Table S1 outlines the naming scheme, as well as the specific impactor velocity values measured by the cameras for each trial. In addition to the

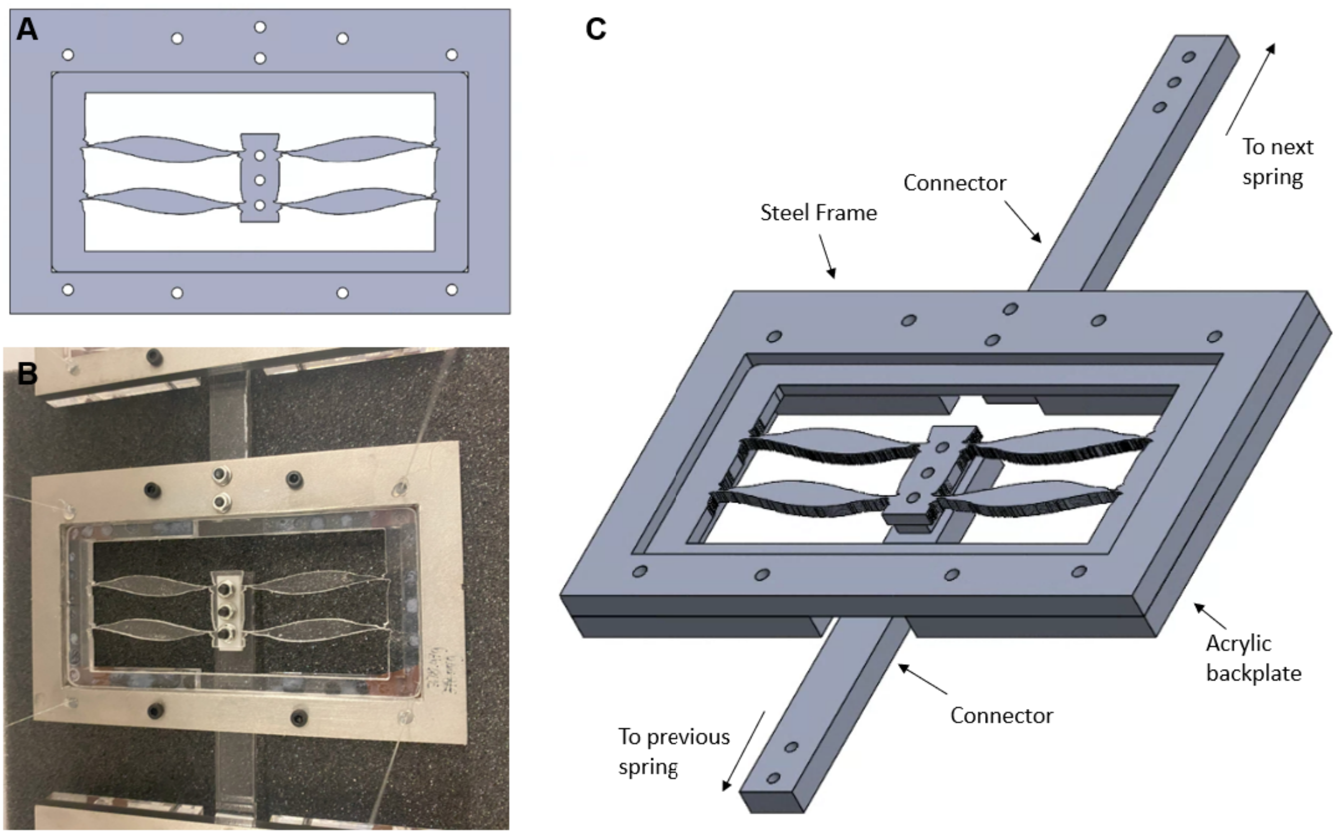

Figure S18: A more detailed breakdown of the unit cell design. A) A CAD model of the nonlinear spring design, embedded inside the frame. B) A photograph of one nonlinear unit cell with connectors attached. C) A CAD model of one nonlinear unit cell including the acrylic backplate and the connectors.

tracked unit cell location collected by the cameras, a vibrometer (specifically, a Polytec PSV 400 laser vibrometer) was pointed at the last unit cell of each chain (unit cell 20), in order to have a second measurement of the velocity of the last unit cell.

Table S1: List of experimentally captured video files and test details (corresponding to data shown in the main text).

Video Name (1-4 for each test)	Spring Tested	Trial Number	Impactor Velocity (m/s)
Main_NonlinTrial1_Vid1.mov through _Vid4.mov	Updated Nonlinear	1	1.38
Main_NonlinTrial2_Vid1.mov through _Vid4.mov	Updated Nonlinear	2	1.36
Main_NonlinTrial3_Vid1.mov through _Vid4.mov	Updated Nonlinear	3	1.37
Main_LinTrial1_Vid1.mov through _Vid4.mov	Updated Linear	1	1.41
Main_LinTrial2_Vid1.mov through _Vid4.mov	Updated Linear	2	1.38
Main_LinTrial3_Vid1.mov through _Vid4.mov	Updated Linear	3	1.39

Two sets of four videos (reduced resolution and sped up via downsampling by 8× to meet file size limitations) corresponding to the data shown in the main text Fig. 5A (nonlinear trial 1) and Fig. 5C (linear trial 1) are included as part of the Supplementary Information. The same naming convention is used as in Supplementary Information Table S1, but with the suffix "_SpedUpLowRes".

Included below are additional experimental data. Figure S19 shows the spatiotemporal kinetic energy maps from all six trials (3 nonlinear and 3 linear). Figure S20 contains the data gathered by the vibrometer (which measured

data only from the last unit cell, unit cell 20, in each chain). We note that the velocities measured by the vibrometer have been processed to account for the angle between the vibrometer and the chain (measured to be 19.35 degrees).

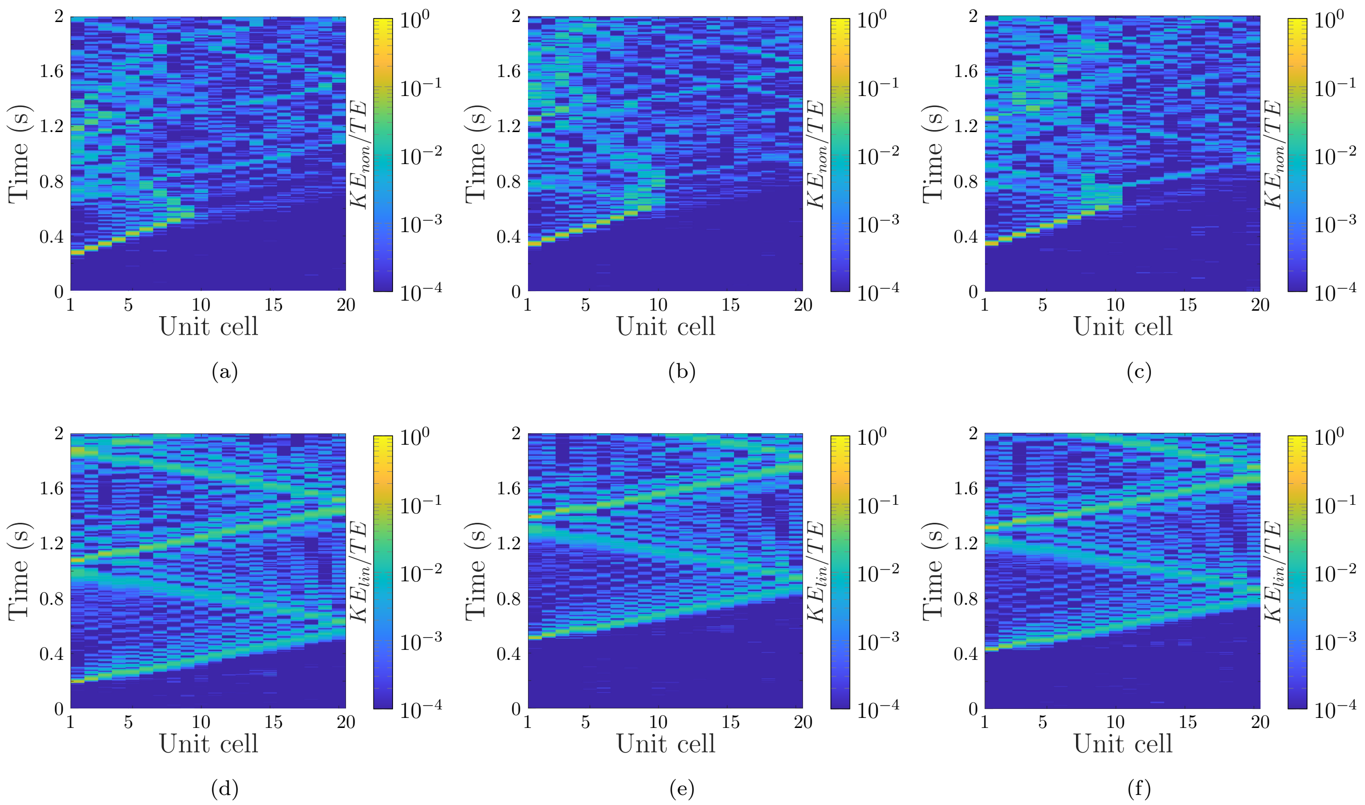

Figure S19: Experimental spatiotemporal kinetic energy response of the chains measured by the cameras, normalized by the input kinetic energy ($0.5MV^2$). (a-c) Nonlinear chain trials 1-3. (d-f) Linear chain trials 1-3. Source data are provided as a Source Data file.

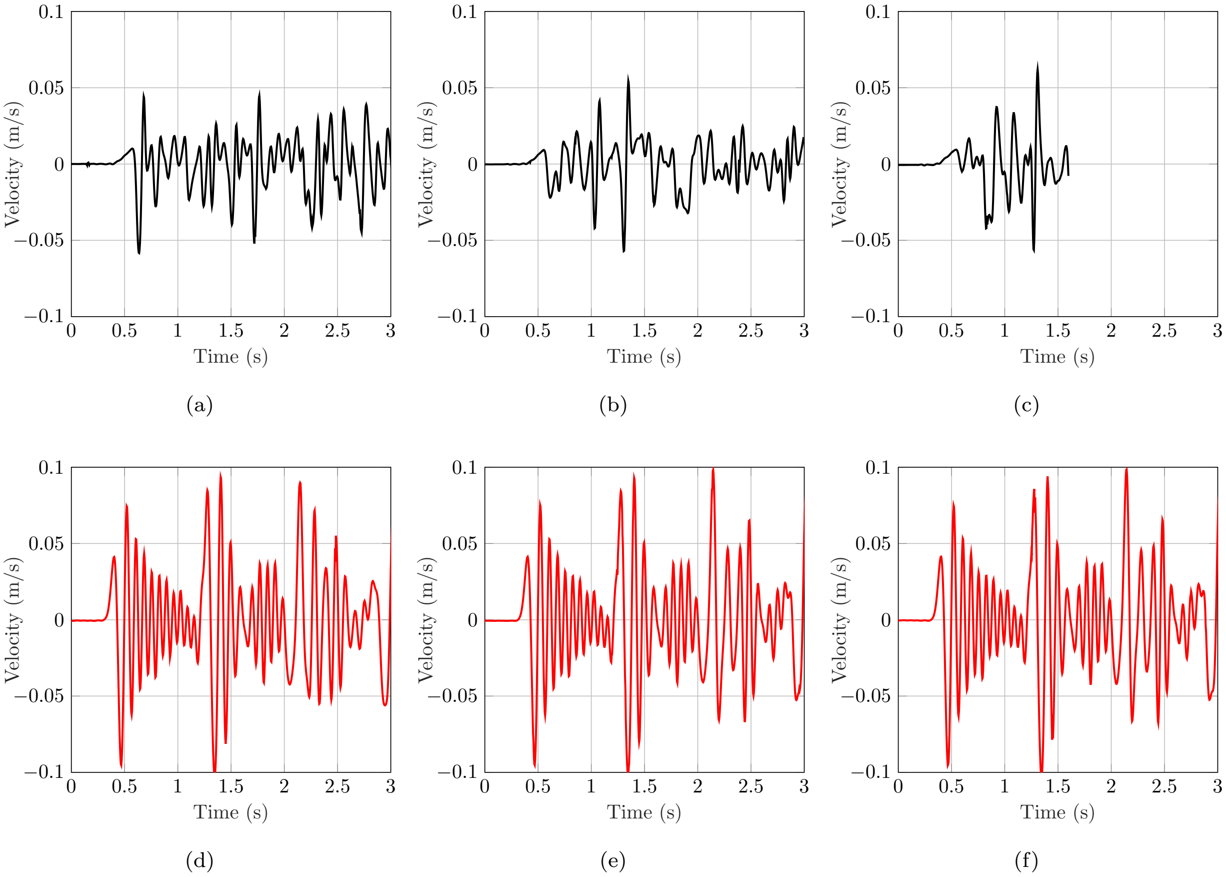

Figure S20: The velocity of the last unit cell of the chain, as processed from the vibrometer data to account for the tilted angle, for (a-c) nonlinear trials 1-3 and (d-f) linear trials 1-3. Source data are provided as a Source Data file.

In Fig. S21(a) and (b), we show the spatiotemporal evolution of the strain at each unit cell, $(x_{i+1} - x_i)/a$, corresponding to Fig. 6A and B in the main text, respectively. The strain being lower than -2.8% indicates that the spring has passed the second stable state, achieving snap-through.

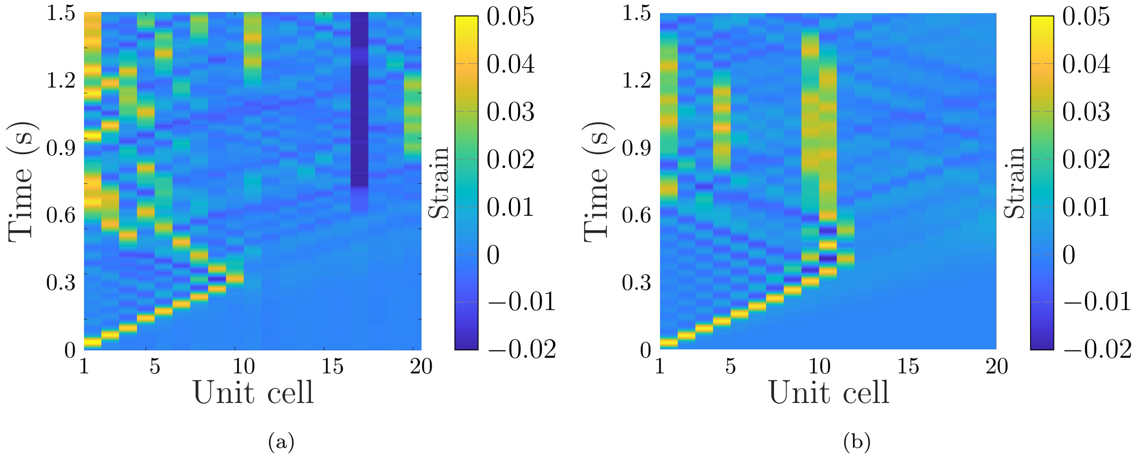

Figure S21: Spatiotemporal evolution of (unit cell scale) strain for the nonlinear system of Fig. 6A and B in the main text. (a) Experiment (corresponding to Fig. 6A in the main text) and (b) simulation (corresponding to Fig. 6B in the main text). When the strain is below -2.8%, the bistable spring snaps through to the second stable state. Source data are provided as a Source Data file.

Note 10: Independence of kinetic energy transmission in the linear DEM chain

A non-intuitive result can be seen where changing the stiffness c_1^* of the linear undamped DEM chain does not affect the amount of kinetic energy transmitted through the chain (Fig. S22), keeping all other parameters constant. In Fig. S22, the time axis is shortened proportional to $\sqrt{c_1^*}$ to account for the faster wave speed.

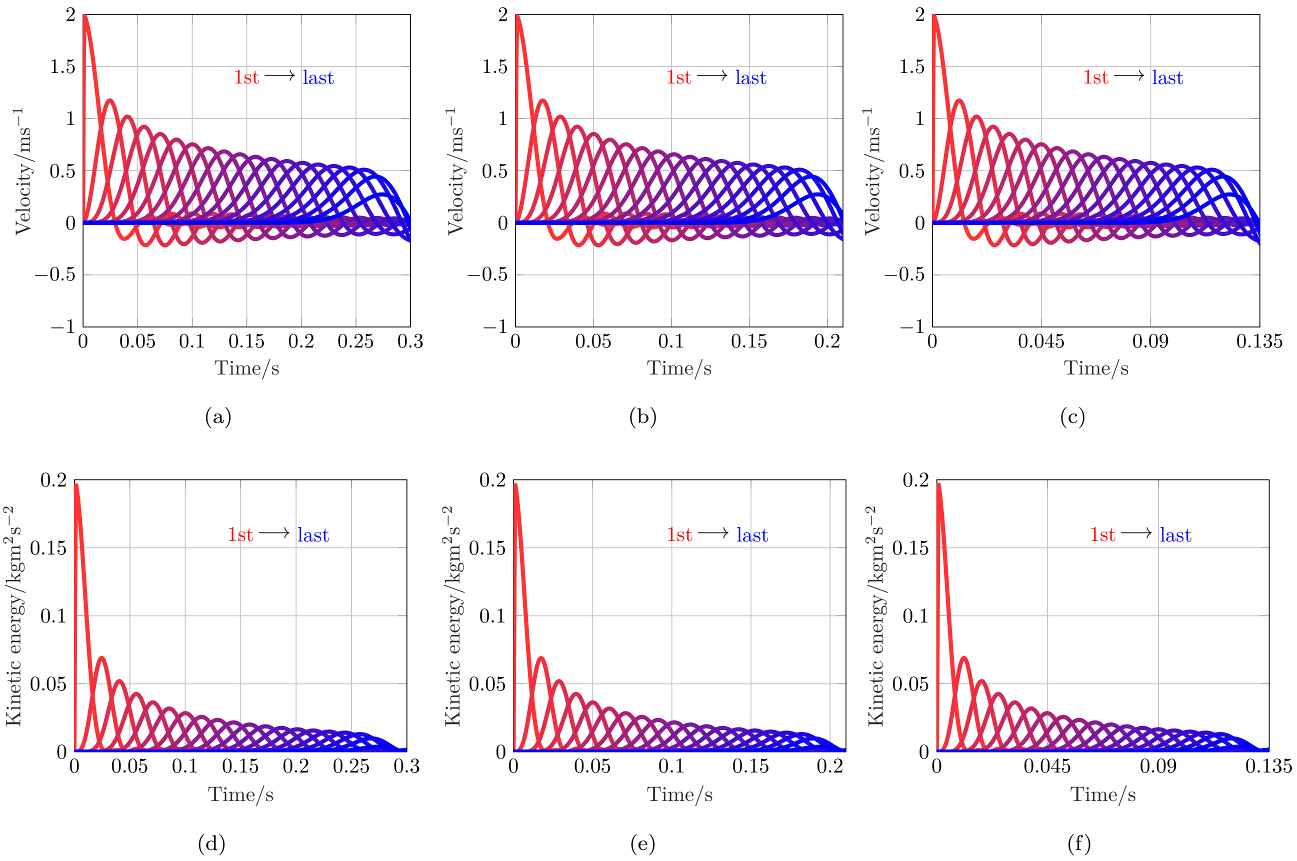

Figure S22: Time-velocity and kinetic energy plots for a DEM-simulated undamped linear chain with different stiffness: (a,d) $c_1^* = 500$ N/m, (b,e) $c_1^* = 1000$ N/m, and (c,f) $c_1^* = 2500$ N/m. All other physical parameters remain constant, $m = 0.1$ kg, $a = 0.1$ m, $M = 0.1$ kg, and $V = 2$ m/s. Source data are provided as a Source Data file.

Note 11: Sensitivity to system and impactor properties, and sharp performance changes upon unit cell snapping for the case of peak transmitted kinetic energy minimization

In Fig. S23(a,b), we show the sensitivity of the $\log_{10}$ of the data in the Fig. 6H in the main text (KE ratio) with respect the impactor conditions M and V, respectively.

We next demonstrate the sharp changes in performance (maximum kinetic energy experienced at the end of the linear chain, divided by the maximum kinetic energy experienced at the end of the nonlinear chain) when an additional unit cell in the chain snaps. Here we consider a slightly different, but still bistable, nonlinear spring from the main text. The parameters for this system are $f(\Delta x) = \Delta x + 87\Delta x^2 + 1778\Delta x^3$, where the linear stiffness is set as $c_1^* = 1.0454$ kN/m, the impactor mass $M_0 = 40$ g, and the length of a unit cell $a = 125$ mm. In Fig. S24, we see very large, periodic jumps in performance (a factor of greater than 10 times) as the impactor velocity is increased even by very

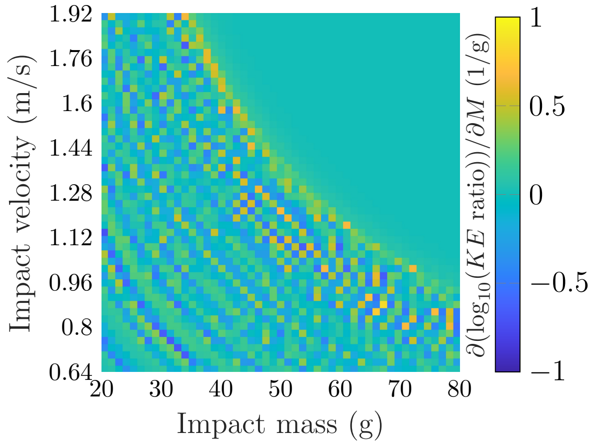

(a)

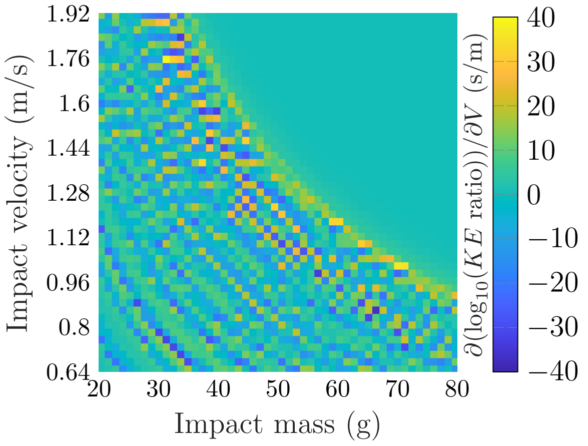

(b)

Figure S23: Performance gradients. Sensitivity of the $\log_{10}$ of the data in Fig. 6H in the main text (KE ratio) with respect to the impactor conditions (c) M and (d) V ($\partial(\log_{10}(KE \text{ ratio}))/\partial M$ and $\partial(\log_{10}(KE \text{ ratio}))/\partial V$, respectively). Source data are provided as a Source Data file.

small amounts of $\sim 3\%$ (~ 0.03 m/s). Here we consider a shorter simulation duration, just over the one-way transit time of a pulse through the material.

Several of the simulations underlying the parameter sweep of Fig. S24 can be seen in Fig. S25, for impactor velocities corresponding to several sequential maximum amplitude points in Fig. S24 ($V = \{1.354, 1.406, 1.454, 1.497\}$ m/s). It can be seen in Fig. S25, that each of these high amplitude points sequentially correspond to one later unit cell in the chain snapping and terminating the motion of the main initial pulse propagating through the system.

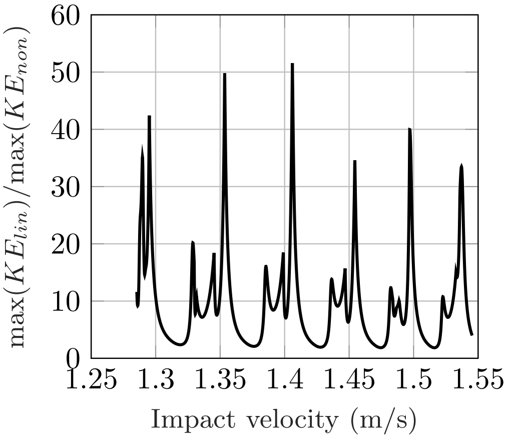

Figure S24: The maximum kinetic energy (linear/nonlinear) versus impactor velocity at the last unit cell using a new set of nonlinear spring coefficients. The new nonlinear mechanical response has a similar behavior to the one used in the experiment with the form of $f(\Delta x) = \Delta x + 87\Delta x^2 + 1778\Delta x^3$. The linear stiffness is set as $c_1^* = 1.0454$ kN/m, the impactor mass is 40 g, and the length of a unit cell is 125 mm. Source data are provided as a Source Data file.

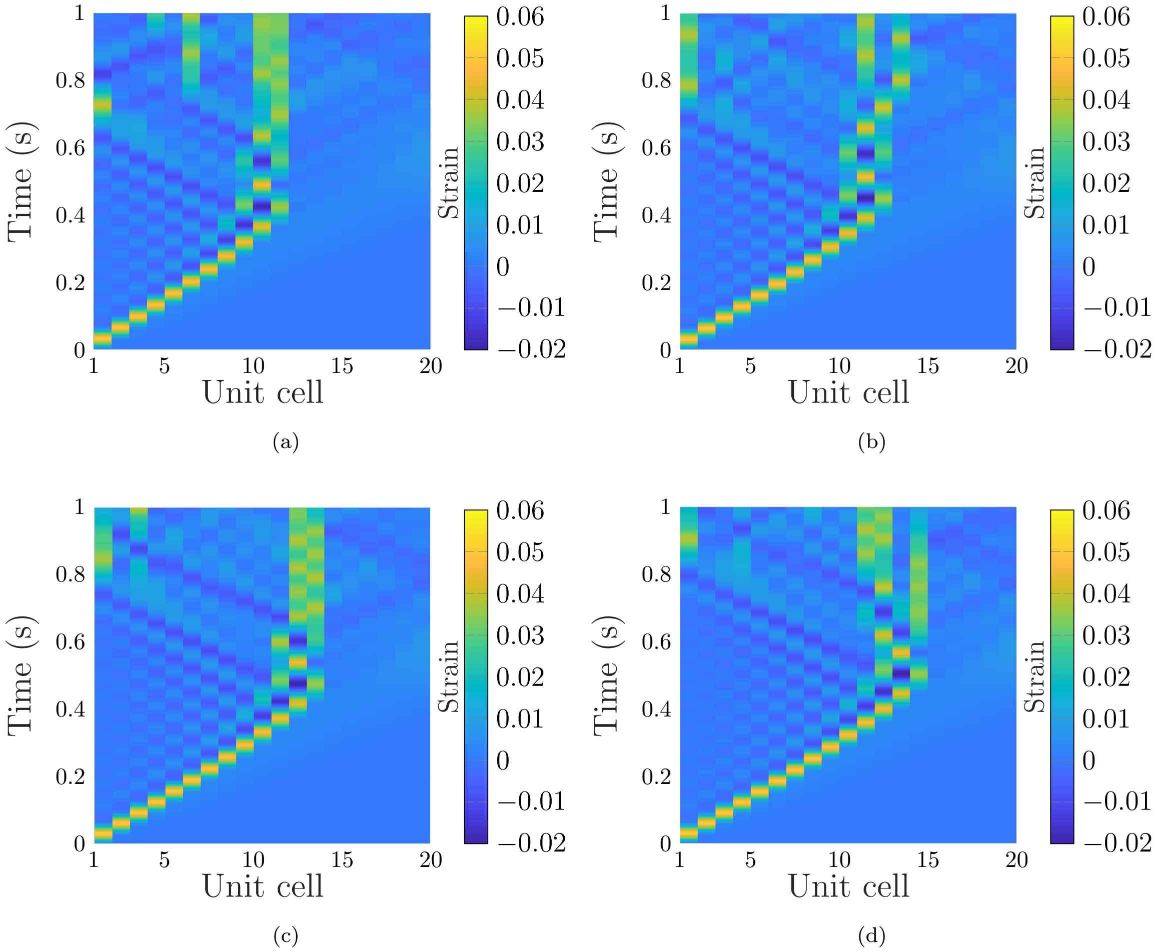

Figure S25: Spatiotemporal evolution of strain in the nonlinear chain for different impactor velocities corresponding to several of the highest amplitude KE ratio peaks in Fig. S24. (a) $V = 1.354$ m/s, (b) $V = 1.406$ m/s, (c) $V = 1.454$ m/s, and (d) $V = 1.497$ m/s. Source data are provided as a Source Data file.

Note 12: Additional experiments with different target coefficients for peak transmitted kinetic energy minimization

Included below are additional experimental data taken from an initial bilevel inverse design attempt (prior to that shown in the main text). In this initial attempt, we did not account for the highly oscillatory nature of the KE performance space with respect to impact conditions (see Fig. 6H in the main text and Fig. S26F below). Thus, small variations in impact velocity had significant effects upon the performance of this initial attempt, highlighting the importance of considering such sensitivity. For the design described in the main text, we took this into account, choosing an optimum with less KE sensitivity to impact conditions. As part of the redesign, we also made the unit cell larger to allow for the manufacturing of finer details in the nonlinear spring, and updated the frame from polycarbonate to stainless steel (which allowed greater stiffness with less volume).

For the initial design, the target coefficients on the unit cell scale were $f(\Delta x) = \Delta x + 94.4\Delta x^2 + 2000\Delta x^3)$, corresponding to $f(\Delta x) = \Delta x_s - 30.2\Delta x_s^2 + 204.8\Delta x_s^3$ on the spring scale (with a value of $a_s = 40mm$), with a final achieved experimental fit of $f(\Delta x) = -0.0003 + \Delta x_s - 28.28\Delta x_s^2 + 193.13\Delta x_s^3$. Figure S26 is the analog for the initial design to the current Fig. 6 in the main text, showing experimental vs. simulation KE maps for the previous experiments, the KE ratio for the previous experiments, and the sensitivity of KE to impact conditions for the previous experiments. We note the lowered average performance in Fig. S26E, as compared to Fig. 6G in the main text.

We note the presence of several nonlinear experimental trials that are excluded from the average of Fig. S26E due

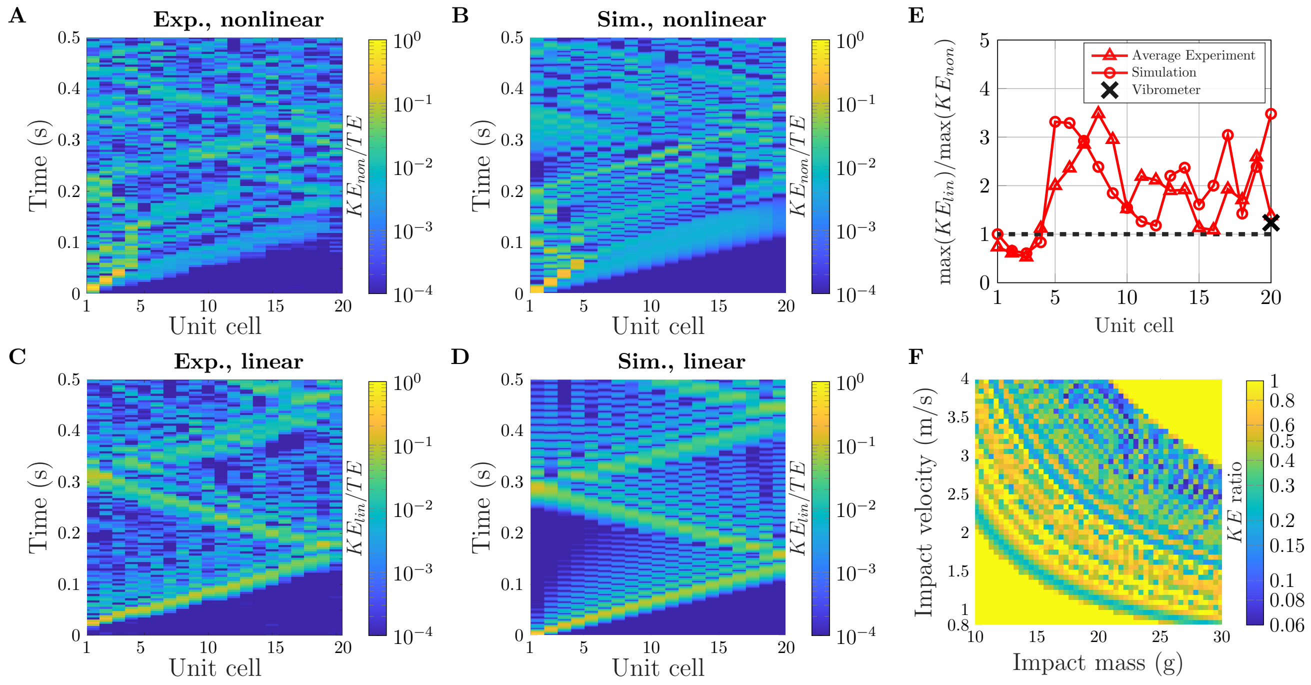

Figure S26: Experimental validation of the previous optimal nonlinear and linear chains, compared with simulation predictions. A-D) Spatiotemporal evolution of kinetic energy in the system (normalized by the input kinetic energy, or initial total energy). The previous nonlinear chain is shown in A (experiment, nonlinear trial 1) and B (simulation), while the previous linear case is shown in C (experiment, linear trial 1) and D (simulation). The experimental spatiotemporal plots include a smoothing of displacement values to assist with noise induced by differentiating the discrete time camera data. E) The maximum kinetic energy (linear/nonlinear) seen at each unit cell for both experiment and simulation. A value greater than 1 indicates superior performance of the nonlinear chain as compared to the linear. The experiment values are the average taken from three experimental trials. The X marks the average experimental value recorded by the vibrometer, which collected data from only the last unit cell. F) The simulated sensitivity of the KE ratio (truncated at 1) to impact conditions for the previous target coefficients. Source data are provided as a Source Data file.

to the onset of anomalous behavior. The videos for these additional trials are included in Table S2). The anomalous behavior included a unexpected increase in observed velocity values as compared to previous trials, particularly in the last unit cell, as well as, in trial 6, an unexpected spike in displacement (accompanied by a unit cell snapping) at unit cell 15, far past the number of unit cells (4-5) expected to snap through to the second stable state. We posit that this behavior was due in part to repeated testing of the system. Multiple trials were run before the data sets herein were collected as experimental procedures and data collection were being refined, and the excluded data were the last three trials run. This was not the case for the updated sets of experiments, presented in the main text, wherein data collection procedures had already been refined through the previous set of experiments. We hypothesize also that the unexpected spike in trial 6 may due to either increased degradation of the spring, or fluctuating impactor velocity pushing the system into a non-ideal condition, or, perhaps, a combination of both.

Figure S27 shows the spatiotemporal kinetic energy maps from six trials (3 nonlinear and 3 linear). Figure S28 contains the data gathered by the vibrometer (which measured data only from the last unit cell, unit cell 20, in each chain). We note that the velocities measured by the vibrometer have been processed to account for the angle between the vibrometer and the chain (measured to be 25.5 degrees).

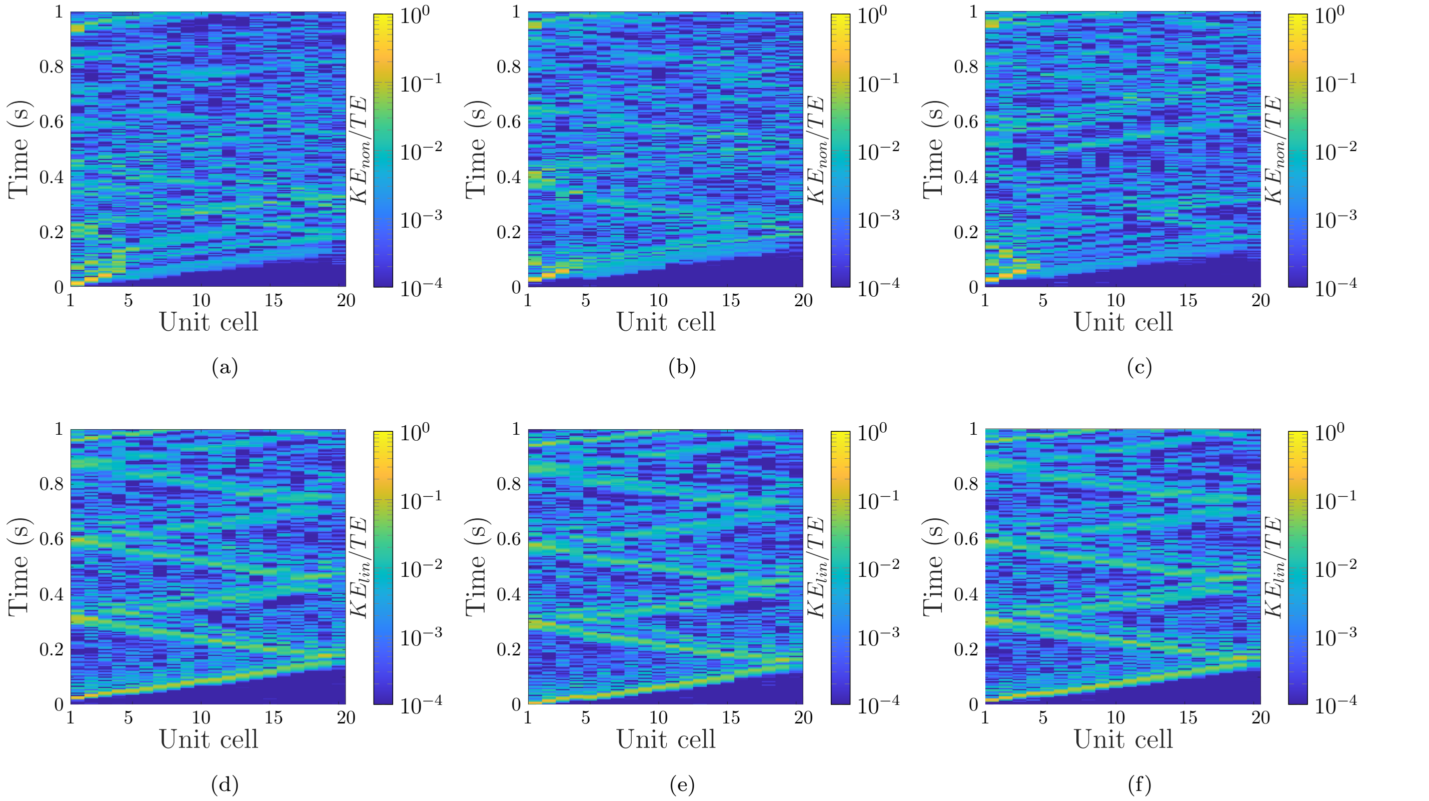

Figure S27: Experimental spatiotemporal kinetic energy response of the chains measured by the cameras, normalized by the input kinetic energy ($0.5MV^2$). (a-c) Nonlinear chain trials 1-3. (d-f) Linear chain trials 1-3. Source data are provided as a Source Data file.

In Fig. S29(a) and (b), we show the spatiotemporal evolution of the strain at each unit cell, $(x_{i+1} - x_i)/a$. When the strain is less than 2.8% the spring has passed the second stable state, achieving snap-through.

The videos of these previous trials are also available in the following online repository: Boechler, Nicholas (2024), "Customizable wave tailoring materials enabled by nonlinear bilevel inverse design 2", Mendeley Data, V2, doi: 10.17632/6bg6hr5kyr.2. The details of each of these files is included in the Table S2 below.

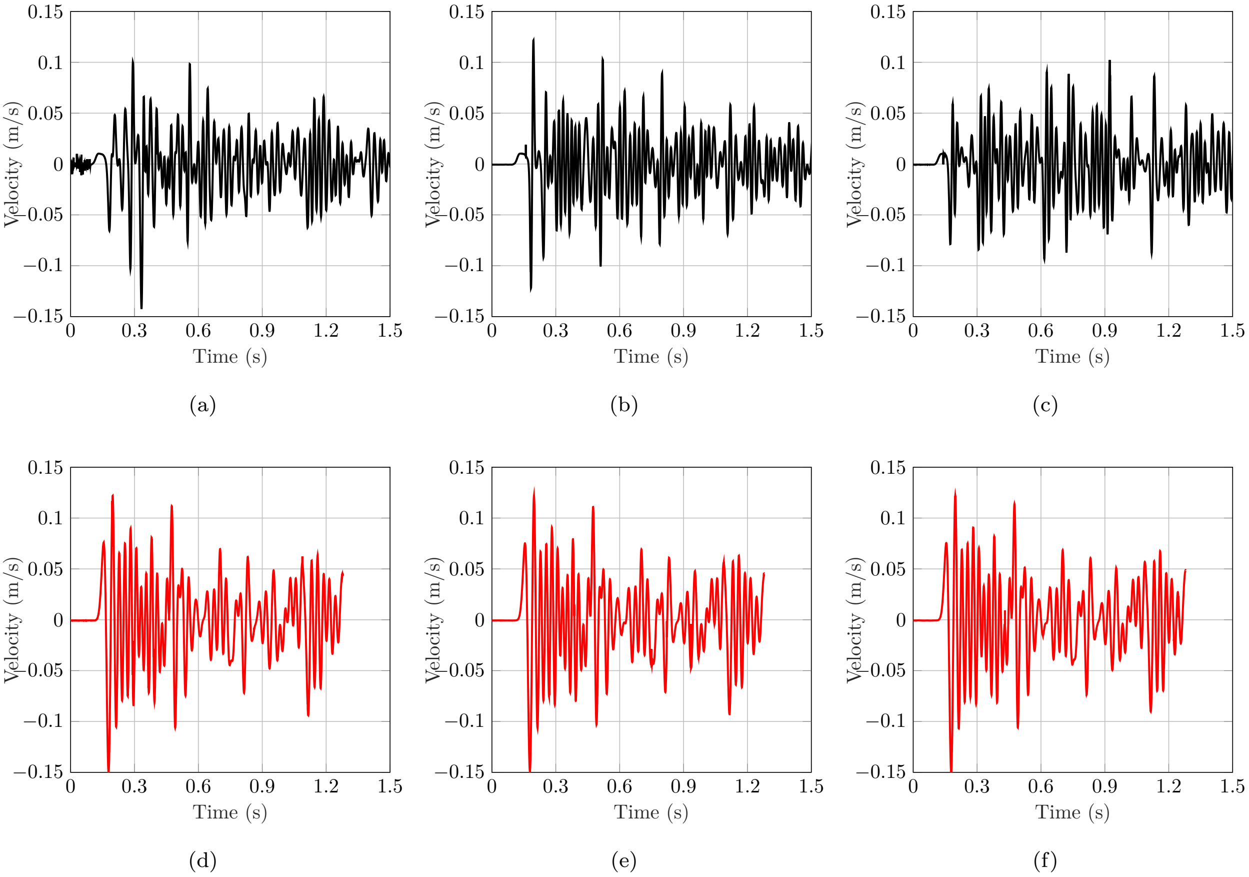

Figure S28: The velocity of the last unit cell of the chain, as processed from the vibrometer data to account for the tilted angle, for (a-c) nonlinear trials 1-3 and (d-f) linear trials 1-3. Source data are provided as a Source Data file.

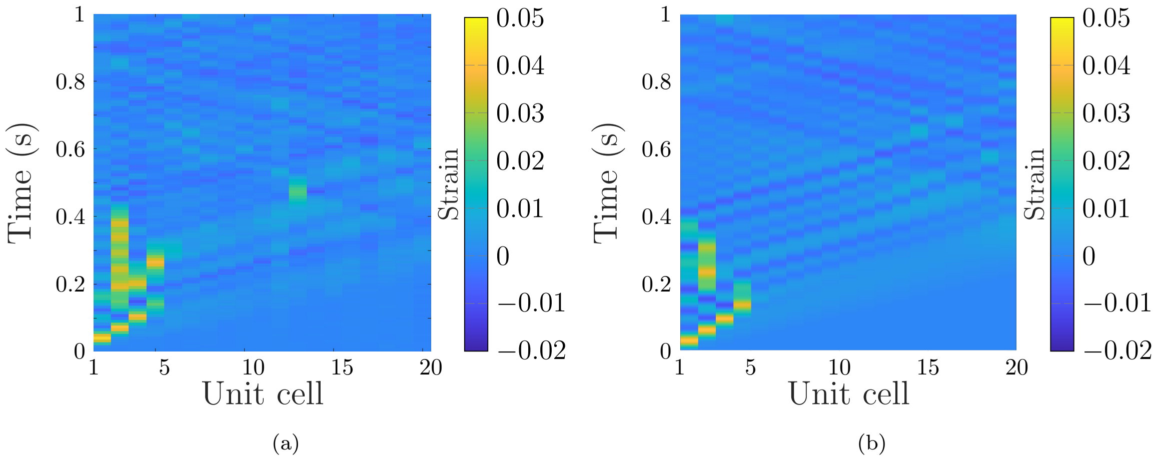

Figure S29: Spatiotemporal evolution of (unit cell scale) strain for the nonlinear system shown in Fig. S27. (a) Experiment and (b) simulation. When the strain is below -2.8%, the bistable spring snaps through to the second stable state. Source data are provided as a Source Data file.

Table S2: List of previous experimentally captured video files and test details.

Video Name<br>(1-4 for each test)	Spring Tested	Trial Number	Impactor Velocity (m/s)
NonlinTrial1_Vid1.MOV through _Vid4.MOV	Nonlinear	1	2.19
NonlinTrial2_Vid1.MOV through _Vid4.MOV	Nonlinear	2	2.10
NonlinTrial3_Vid1.MOV through _Vid4.MOV	Nonlinear	3	2.14
NonlinTrial4_Vid1.MOV through _Vid4.MOV	Nonlinear (unused)	4	2.16
NonlinTrial5_Vid1.MOV through _Vid4.MOV	Nonlinear (unused)	5	2.14
NonlinTrial6_Vid1.MOV through _Vid4.MOV	Nonlinear (unused)	6	2.11
LinTrial1_Vid1.MOV through _Vid4.MOV	Linear	1	2.23
LinTrial2_Vid1.MOV through _Vid4.MOV	Linear	2	2.20
LinTrial3_Vid1.MOV through _Vid4.MOV	Linear	3	2.16

References

[1] Hertz, H. Ueber die berührung fester elastischer körper. (1882).
